# Changes in the Epidemiology and Outcomes of Emergency Department Presentations for COVID‐19 Infection, 2020 Through 2022, New South Wales, Australia: A Population‐Based Record Linkage Study

**DOI:** 10.1111/irv.70246

**Published:** 2026-03-06

**Authors:** Kishor K. Paul, Nectarios Rose, Sandra Ware, Michael M. Dinh, David J. Muscatello

**Affiliations:** ^1^ School of Population Health University of New South Wales Sydney Australia; ^2^ Aeromedical Operations New South Wales Ambulance Sydney Australia; ^3^ Emergency Department Royal Prince Alfred Hospital Sydney Australia

**Keywords:** COVID‐19 outcome, linked data, New South Wales, SARS‐CoV‐2

## Abstract

**Introduction:**

Despite substantial human and financial resources invested in managing the pandemic, Australia lacks information on COVID‐19 epidemiology and health service use. This study aimed to describe the epidemiology of emergency department (ED) patients during the first 2 years of the pandemic in New South Wales (NSW), Australia (population ~8.2 million).

**Methods:**

NSW respiratory infection‐related ED presentations were probabilistically linked with ambulance, admitted patient, notifiable condition, and death databases. ED patients with a linked COVID‐19 infection notification were included. The study spanned 2020 through 2022 and was divided into three phases according to the dominant virus variant: pre‐Delta, Delta, and Omicron.

**Results:**

Of the 92,331 ED presentations with a linked COVID‐19 infection during the study period, most occurred during the Omicron phase (81%, 782%, 89%), followed by 9514 (10%) in the Delta and 1035 (1%) in the pre‐Delta phase. From the Delta to the Omicron phase, there was a substantial increase in the incidence of ED presentations among < 10 and ≥ 80‐year‐olds, with a 28‐fold and 20‐fold increase, respectively. Similarly, inpatient admissions increased 8‐fold and 17‐fold in these two age groups between the two phases. Over the same period, regional NSW residents experienced a 31‐fold and 15‐fold increase in ED presentations and inpatient admissions, while Greater Sydney residents experienced a 6‐fold and 3‐fold increase, respectively.

**Conclusions:**

The study highlights the epidemiological shifts during the pandemic in NSW, especially the Omicron phase with marked increases in presentations and admissions, especially in the oldest adults and in children, and spread to regional areas.

## 1 Introduction

A novel coronavirus, SARS‐CoV‐2, causing coronavirus disease 2019 (COVID‐19) emerged in China in 2019 and spread quickly to become a pandemic, pushing healthcare systems to capacity [1, 2, 3]. In Australia, emergency departments (EDs) provided frontline hospital care during the pandemic. The epidemiology of ED presentations of Australian patients with SARS‐CoV‐2 infection has only previously been described to August 2020 and from only 12 EDs [4, 5].

In New South Wales (NSW), Australia's most populous state (~8.2 million), COVID‐19‐related hospital admissions, including intensive care unit (ICU) stays and ventilation needs were described to May 2020 [6]. That analysis used linked data from the Notifiable Conditions Information Management System (NCIMS) and the NSW Admitted Patient Data Collection (APDC) databases. However, the study did not evaluate the use of health resources needed before inpatient admission, including ambulance services or ED presentations. Another study conducted between June and October 2021 documented the severity and clinical spectrum of COVID‐19 in children but included data from only two children's hospitals in Sydney [7].

From January 2020 to November 2021, the COVID‐19 epidemic in Australia was primarily caused by the original, Beta, and Delta variants, with community transmission strongly controlled by public health interventions including border closures, contact tracing, testing, isolation, and quarantine, and hygiene and movement restrictions [8, 9]. Since the relaxation of control measures from December 2021, NSW and Australia have experienced multiple waves of COVID‐19 infection with the coincident emergence of the Omicron variant [10]. The magnitude and duration of these waves varied due to changing public health interventions and population‐level immunity from prior infections and vaccination programs [11].

Australia experienced a substantial health service burden due to the pandemic and invested considerable human and financial resources to manage it [2, 12], but lacks information on the epidemiology of ED outcomes of COVID‐19 infections. Improved description of the epidemiology can inform preparation for future epidemics and pandemics. We used a rich, linked healthcare database to describe the evolving epidemiology and short‐term outcomes of COVID‐19 patients seeking care in public hospital EDs in NSW over three epidemic periods to the end of 2022.

## 2 Methods

### 2.1 Study Design, Setting, and Study Period

This was a descriptive study conducted using probabilistic record linkage in NSW, Australia, for the period 2020 through 2022.

### 2.2 Data Source

Data were extracted from the anonymized PEARL (Pandemic and Epidemic Assessment of Risk using Linked data) database established for research on acute respiratory infection epidemics in NSW. The PEARL database includes a cohort of patients presenting to any ED participating in the NSW ED Data Collection (EDDC) and who were assigned an ED diagnosis that could be related to an acute respiratory infection. The EDDC receives reports on episodes of care from all public hospital EDs across the state, except one recently established urban public or any of the few private hospital EDs in NSW.

The principal ED diagnosis, which can indicate either a symptom, syndrome, or specific condition, was used for including patients in the PEARL database. Given the variety of ED electronic medical record systems used in NSW, the diagnoses may be provided as International Classification of Diseases codes, revisions 9 (ICD‐9) or 10 (ICD‐10), or as Systematized Nomenclature of Medicine, Clinical Terminology (SNOMED‐CT) identifiers.

The PEARL database includes ED patients receiving a diagnosis falling into any of the following categories: pneumonia, influenza or influenza‐like illness, coronavirus infection, bronchiolitis, other or unspecified acute respiratory illness, fever or unspecified infection, cough, breathing or respiratory problems (excluding asthma), and sepsis. Included diagnosis codes for each category are available in the supplementary material of Muscatello et al. [13].

Each ED record was probabilistically linked, using privacy‐preserving methods [14], to records for the same person from the NSW Ambulance medical record and dispatch databases, Admitted Patient Data Collection (APDC), Notifiable Conditions Information Management System (NCIMS), and death registrations from the state Registry of Births, Deaths, and Marriages (RBDM) [13]. The EDDC receives reports on episodes of care from all public hospital EDs across the state, except one recently established urban public or any of the few private hospital EDs in NSW. The NSW APDC reports on inpatient episodes of care from all public and private hospitals in the state and includes ICU and ventilation status and duration. Private hospital inpatient records were only available to June 2022. The NCIMS includes registrations of confirmed and probable SARS‐CoV‐2 diagnoses reported to NSW Public Health Units under public health legislation. Confirmed diagnoses of SARS‐CoV‐2 required laboratory definitive evidence, while probable diagnoses were based on rapid antigen tests that were reported using a government services app commencing in early 2022 and continuing to the end of the study period [15].

### 2.3 Study Inclusion and Exclusion Criteria

Patients in the PEARL database presenting during the study period and residing in, and acquiring COVID‐19 in, NSW were included. Only community‐acquired cases were included, defined as a linked SARS‐CoV‐2 result from NCIMS with an earliest available specimen collection date during the 28 days prior to 3 days after ED arrival. Specimens collected beyond 3 days of ED arrival were excluded to avoid including hospital‐acquired infection. The first ED visit by an individual within any 28‐day period was considered the index ED visit for SARS‐CoV‐2 infection. Subsequent ED presentations within this period were considered associated with initial infection. Linked inpatient and death outcomes within 28 days of the index ED visit, and any ambulance transport during 1 day prior until 28 days after the index ED visit were included.

### 2.4 Definitions

The study period was divided into three pandemic phases: pre‐Delta (on or before 31 May 2021), Delta (1 June through 30 November 2021), and Omicron (1 December 2021 through 31 December 2022). The pre‐Delta phase in NSW was characterized by sporadic outbreaks contained by border measures and testing, tracing, isolation, and quarantine (TTIQ). The Delta phase, following a single case detection of the higher transmissibility variant in June 2021, was a larger outbreak mitigated by TTIQ supplemented with increasing COVID‐19 vaccination coverage, movement restrictions, and stay‐at‐home orders [16]. The Omicron period coincided with relaxation of control measures and arrival of the original Omicron variant. The Omicron phase was ongoing at the study end date and included several epidemic waves linked to multiple Omicron sub‐variants [17, 18, 19].

The variables and their categories considered in this study included age (10‐year age groups to 79 years and ≥ 80 years), sex, region of residence (Greater Sydney, rest of NSW), preferred language (English, other), country of birth (Australia, other), mode of arrival to the ED (ambulance, other), and the number of ED and admission episodes experienced by the patient during the 28‐day follow‐up period. Ambulance transport includes inter‐ or intra‐hospital transfer. Urgency of medical assessment for the patient's condition, assessed at ED triage according to the Australasian Triage Scale, is standardized in Australia into five categories: 1 = Resuscitation, 2 = Emergency, 3 = Urgent, 4 = Semi‐urgent, and 5 = Less‐urgent [20]. The ED record and any linked admission or death records were used to assign a “worst outcome” variable to allocate the patient's status at 28 days following the index ED arrival. Worst outcome was categorized as: death, ICU admission, inpatient admission, and ED only. For patients admitted to the ICU, data on hours spent in the ICU and ventilatory support status, including hours on ventilatory support, were also available.

### 2.5 Analysis

We conducted descriptive analysis of the included ED presentations with comparison over the three phases of the epidemic. Due to the large data size, we did not calculate chi‐square statistics for differences between groups but focused on practical significance. Mid‐year population estimates within each phase were used for incidence rates [21].

## 3 Results

### 3.1 ED Presentations

**FIGURE 1:**
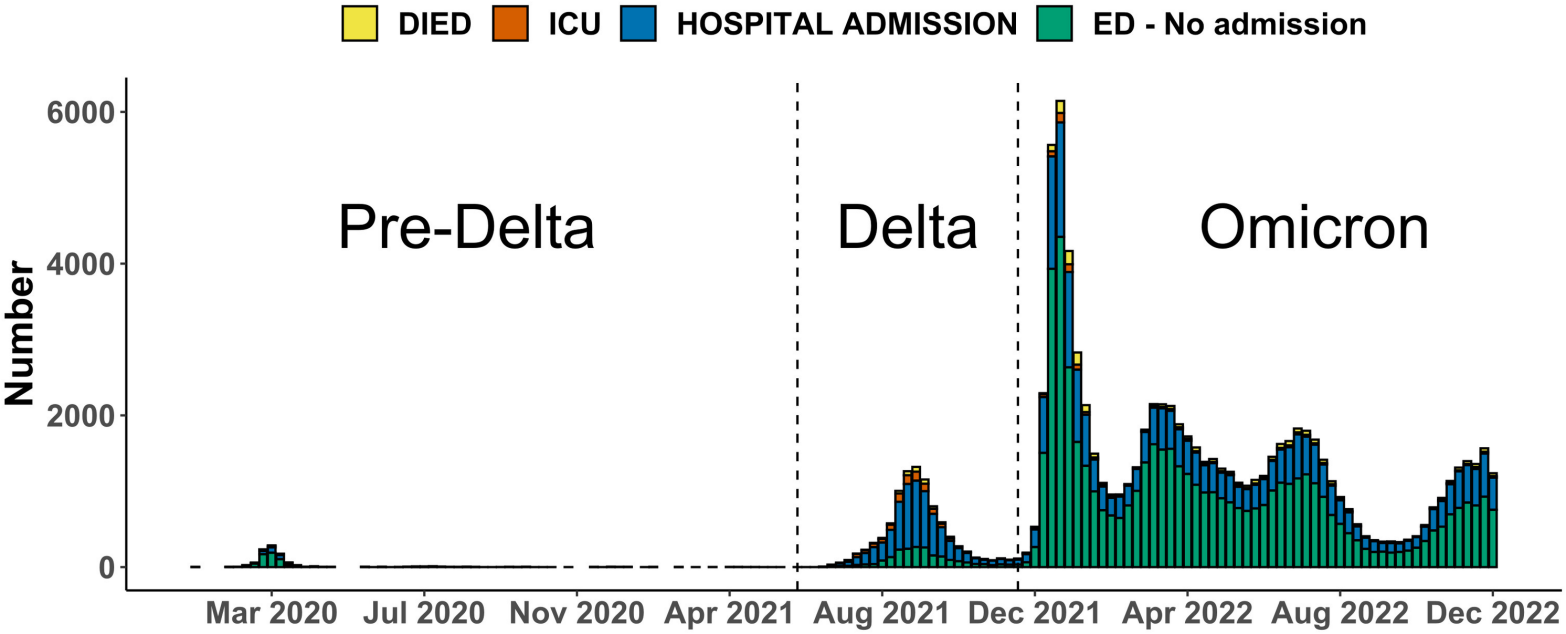
Weekly number of emergency department (ED) presentations with a linked SARS‐CoV‐2 infection, by worst 28‐day outcome; Excludes information from one recently opened public ED service in Sydney and four private ED services in NSW. Epidemic phases: pre‐Delta (before June 1, 2021), Delta (June 1, 2021–November 30, 2021), and Omicron (December 1, 2021–December 31, 2022). As required by the data provider, nonzero counts < 5 were set to 5 to avoid patient information disclosure risk.

During January 2020 through December 2022, a total of 91,546 patients made 92,331 index ED presentations with an included diagnosis to 168 of the 176 EDs and had a confirmed or probable SARS‐CoV‐2 infection. Most index ED presentations occurred during the Omicron phase (81,782, 89%) and the fewest in the pre‐Delta phase (1035, 1%), while 9514 (10%) occurred in the Delta phase (Figure 1, Table 1). Overall, the median age of patients was 39 years (interquartile range (IQR) 18–65 years). During the Omicron phase, the median age reduced to 38 (IQR 14–67) from 52 (IQR 33–66) years in the pre‐Delta phase and 42 (IQR 28–59) years in the Delta phase (Table 1).

**TABLE 1:** Characteristics and 28‐day outcomes of index emergency department (ED) presentations with a linked SARS‐CoV‐2 infection.

Characteristics	Pre‐Delta (*N* = 1035) *n* (%)	Delta (*N* = 9514) *n* (%)	Omicron (*N* = 81,782) *n* (%)	Total (*N* = 92,331) *n* (%)
Age in years^a^	52 (33, 66)	42 (28, 59)	38 (14, 67)	39 (18, 65)
Age groups (years)				
< 10	15 (1)	674 (7)	18,474 (23)	19,163 (21)
10–19	29 (3)	611 (6)	5300 (6)	5940 (6)
20–29	160 (16)	1448 (15)	9222 (10)	10,830 (12)
30–39	146 (14)	1688 (18)	9347 (11)	11,181 (12)
40–49	127 (12)	1525 (16)	7420 (9)	9072 (10)
50–59	172 (17)	1351 (14)	6961 (8)	8484 (9)
60–69	185 (18)	1045 (11)	7068 (9)	8298 (9)
70–79	140 (14)	690 (7)	8081 (10)	8911 (10)
80+	61 (6)	482 (5)	9909 (13)	10,452 (11)
Sex^b^				
Female	532 (51)	4831 (51)	43,451 (53)	48,814 (53)
Male	503 (49)	4682 (49)	38,322 (47)	43,507 (47)
Any ambulance transport	276 (27)	7073 (74)	24,338 (30)	31,687 (34)
Location of residence				
Greater Sydney	847 (82)	8566 (90)	52,421 (64)	61,834 (67)
Regional NSW	188 (18)	948 (10)	29,361 (36)	30,497 (33)
Country of birth				
Australia	623 (60)	4729 (50)	56,765 (69)	62,117 (67)
Other	412 (40)	4785 (50)	25,017 (31)	30,214 (33)
Preferred language				
English	908 (88)	6734 (71)	68,571 (84)	76,213 (82)
Other	127 (12)	2780 (29)	13,211 (16)	16,118 (18)
Triage urgency on index presentation^c^				
1‐Resuscitation	13 (1)	180 (2)	767 (1)	960 (1)
2‐Emergency	113 (11)	2600 (27)	14,824 (18)	17,537 (19)
3‐Urgent	227 (22)	4686 (49)	35,929 (44)	40,842 (44)
4‐Semi‐urgent	221 (21)	1582 (17)	25,578 (31)	27,381 (30)
5‐Less‐urgent	460 (44)	460 (5)	4673 (6)	5593 (6)
ED diagnosis category				
Coronavirus infection	581 (56)	8447 (89)	58,705 (72)	67,733 (73)
Other or unspecified ARI	173 (17)	343 (4)	6604 (8)	7459 (8)
Fever or infection due to unspecified cause	111 (11)	194 (2)	7154 (9)	7120 (8)
Breathing or respiratory problems, potentially ARI related, non‐asthma	80 (7)	296 (3)	4501 (6)	4877 (5)
Cough	34 (3)	54 (1)	1596 (2)	1684 (2)
Pneumonia	32 (3)	127 (1)	1481 (2)	1640 (2)
Influenza, influenza like illness	21 (2)	13 (< 1)	568 (1)	602 (1)
Sepsis due to unspecified cause or possible ARI	5^d^ (< 1)	25^d^ (< 1)	665 (1)	694 (1)
Bronchiolitis	0^d^ (0)	15^d^ (< 1)	508 (1)	522 (1)
Any inpatient admission	406 (39)	7253 (76)	26,816 (33)	34,475 (37)
ICU admission	96 (9)	1128 (12)	1679 (2)	2903 (3)
Death	31 (3)	378 (4)	2168 (3)	2577 (3)

Abbreviations: ARI, acute respiratory infection; NSW, New South Wales.

^a^ Median and interquartile range (IQR).

^b^ Nonbinary or not available for 10 (0.01%) patients.

^c^ Not available for 18 (0.02%) patients.

^d^ The data provider required small cell counts (< 5) to be masked to avoid patient information disclosure risk, so these table cells were rounded to the nearest multiple of 5. To avoid determination of the counts in these cells by subtraction, cells adjacent to the small counts were also rounded to the nearest 5.

Children aged < 10 years accounted for the highest proportion, 21% (19,163) overall of index ED presentations. During the pre‐Delta phase, children aged < 10 years represented 1% [15] of index ED presentations, increasing to 7% (674) during the Delta phase, and 23% (18,474) during the Omicron phase. Overall, females accounted for 53% (48,814) of all index ED presentations, and the proportion varied little by phase. Just over one third (34%, 31,687) of index ED presentations had at least one associated ambulance transport event. The proportion was 27% (276) during the pre‐Delta phase, 30% (24,338) during Omicron, and 74% (7073) during the Delta phase (Table 1).

Overall, one‐third (30,497) of all index ED presentations were among residents of regional NSW. The proportions were 18% (188) and 10% (948) for the pre‐Delta and Delta phases, respectively, and 36% (29,361) during Omicron. Two‐thirds (62,117) of patients were born in Australia, and 82% (76,213) preferred speaking English. The proportion born in Australia was one‐half (4729) during the Delta phase but was just over two‐thirds (69%) during the Omicron phase (Table 1).

The coronavirus infection ED diagnosis grouping accounted for almost three‐quarters of index presentation diagnoses (73%, 67,733). The proportion was 56% (581) during the pre‐Delta phase, increasing substantially to 89% (8447) during Delta, and declining to 72% (58,705) during the Omicron phase. The next most frequent diagnosis categories were “fever or unspecified infection” and “other or unspecified acute respiratory illness”, with each accounting for 8% overall.

Overall, just over one third (37%, 34,475) of index presentations had at least one inpatient admission within 28 days, although the proportion was just over three quarters (76%) during the Delta phase. About 3% (2903) of index presentations had an ICU admission overall. This proportion was 12% (1128) during the Delta, 9% (96) during the pre‐Delta and 2% (1679) during the Omicron phase. Three per cent of index ED presentations were followed by death within 28 days during the pre‐Delta and Omicron phases, increasing to 4% during the Delta phase (Table 1).

### 3.2 ED Presentations With Inpatient Admission Within 28 Days

The median age of patients with any inpatient admission after the index ED presentation across all three phases of the epidemic was 62 years (IQR 33–79 years) but was 45 years (IQR 30–61 years) during the Delta phase. While adults aged between 20 and 69 years accounted for a majority of inpatient admissions during the pre‐Delta (63%, 256) and Delta (73%, 5324) phases, they accounted only for 37% (9596) during the Omicron phase (Table 2). Children aged < 10 years accounted for 15% (3996) of inpatient admissions during Omicron compared with 2% [7] and 7% (494) during the pre‐Delta and Delta phases, respectively. Similarly, ≥ 80‐year‐olds accounted for 29% (7655) of inpatient admissions during the Omicron phase compared to 13% (51) and 6% (447) admissions during the pre‐Delta and Delta phases, respectively.

**TABLE 2:** Characteristics and outcomes of emergency department (ED) presentations with a linked SARS‐CoV‐2 infection and inpatient admission within 28 days of index presentation.

Characteristics	Pre‐Delta (*N* = 406) *n* (%)	Delta (*N* = 7253) *n* (%)	Omicron (*N* = 26,816)*n* (%)	Total (*N* = 34,475)*n* (%)
Median age (years)^a^	62 (42, 73)	45 (30, 61)	68 (34, 82)	62 (33, 79)
Age groups (years)				
< 10	7 (2)	494 (7)	3996 (15)	4497 (13)
10–19	10 (2)	377 (5)	682 (3)	1069 (3)
20–29	25 (6)	951 (13)	1305 (5)	2281 (7)
30–39	45 (11)	1194 (17)	1555 (6)	2794 (8)
40–49	42 (10)	1182 (16)	1536 (6)	2760 (8)
50–59	60 (15)	1115 (15)	2039 (8)	3214 (9)
60–69	84 (21)	882 (12)	3161 (12)	4127 (12)
70–79	82 (20)	611 (8)	4887 (18)	5580 (16)
80+	51 (13)	447 (6)	7655 (29)	8153 (24)
Sex^b^				
Female	193 (48)	3627 (50)	12,822 (48)	16,642 (48)
Male	213 (52)	3625 (50)	13,994 (52)	17,832 (52)
Any ambulance transport	211 (52)	5695 (79)	14,400 (54)	20,306 (59)
Location				
Greater Sydney	323 (80)	6698 (92)	18,517 (69)	25,538 (74)
Regional NSW	83 (20)	555 (8)	8299 (31)	8937 (26)
Inpatient LOS (days)^a^	10 (4, 18)	8 (3, 14)	5 (2, 10)	6 (2, 11)
LOS in ICU (hours)^a^	144 (66, 362)	140 (54, 314)	82 (36, 190)	100 (43, 240)
Any ventilation	49 (12)	418 (6)	483 (2)	950 (3)
Duration (hours)^a^, ^c^	358 (223, 802)	407 (242, 694)	246 (113, 426)	318 (168, 544)
Death	29 (7)	356 (5)	1918 (7)	2303 (7)

Abbreviations: ARI: acute respiratory infection; LOS: cumulative ward length of stay, excluding ED episodes; NSW: New South Wales; SARS: Severe acute respiratory syndrome.

^a^ Median and interquartile range (IQR).

^b^ Nonbinary or not available for 10 patients.

^c^ Cumulative duration of ventilation among those ventilated.

Almost four‐fifths (79%, 5695) of inpatients had an ambulance transport during the Delta phase, compared with 59% (20,306) overall. Almost one‐quarter (24%, 96) of inpatients were admitted to an ICU during the pre‐Delta phase, compared with 16% (1128) and 6% (1679) during the Delta and Omicron phases, respectively. Ventilation use was 12% (49), 6% (418), and 2% (483) during the pre‐Delta, Delta, and Omicron phases, respectively (Table 2).

Overall, death, in or out of hospital, within 28 days occurred in 7% (2303) of inpatients, but was 5% (356) during the Delta phase (Table 2). Of the overall deaths, 345 (15%) occurred out of hospital based on being alive at hospital discharge and presence of a linked death registration. No deaths were recorded among inpatients < 10‐year olds during pre‐Delta and Delta phases, while < 5 deaths were recorded during Omicron phase.

### 3.3 Incidence of ED Presentations and Inpatient Admissions

**FIGURE 2:**
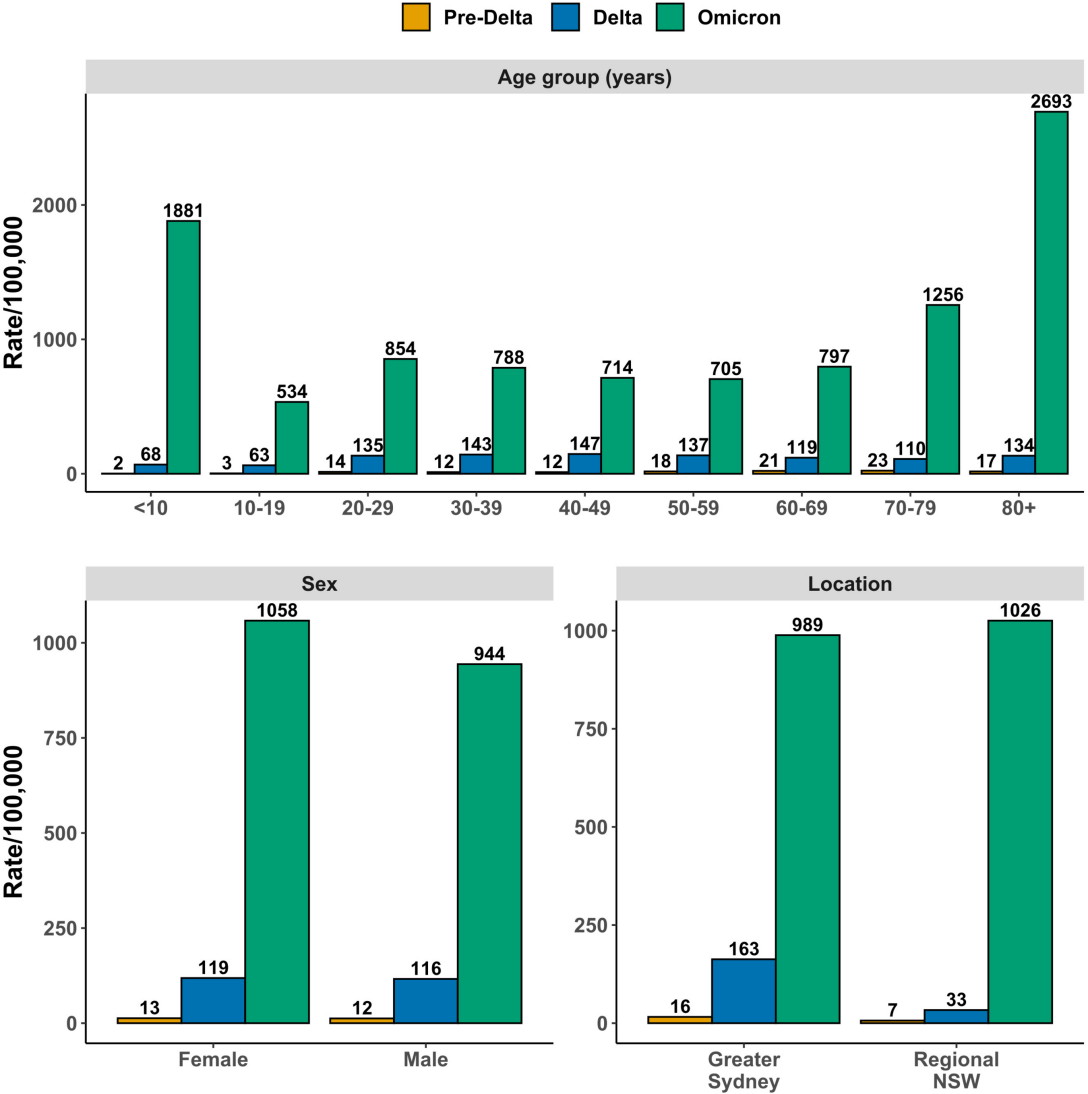
Incidence per 100,000 population of emergency department (ED) presentations with a linked SARS‐CoV‐2 infection, by age, sex, and location of residence.

The incidences of index ED presentations during the pre‐Delta, Delta, and Omicron phases were 13, 117, and 1002 per 100,000, respectively. Children aged < 10 years had the lowest incidence of 2 per 100,000 during the pre‐Delta phase and the second lowest at 68 per 100,000 during the Delta phase, increasing 28‐fold to 1881 per 100,000 during the Omicron phase. During the Omicron phase, ≥ 80‐year‐olds had the highest incidence (2693 per 100,000), and a 20‐fold increase from the Delta phase (Figure 2).

During the Omicron phase, the incidence of female index ED presentations was slightly higher than that of male ED presentations (1058 vs. 944 per 100,000), despite being comparable during other phases. Compared with the Omicron phase, incidence was much higher among Greater Sydney residents during the pre‐Delta and Delta phases than regional residents. During the Omicron phase, incidence was slightly lower among Sydney residents (989 vs. 1026 per 100,000 population) (Figure 2).

**FIGURE 3:**
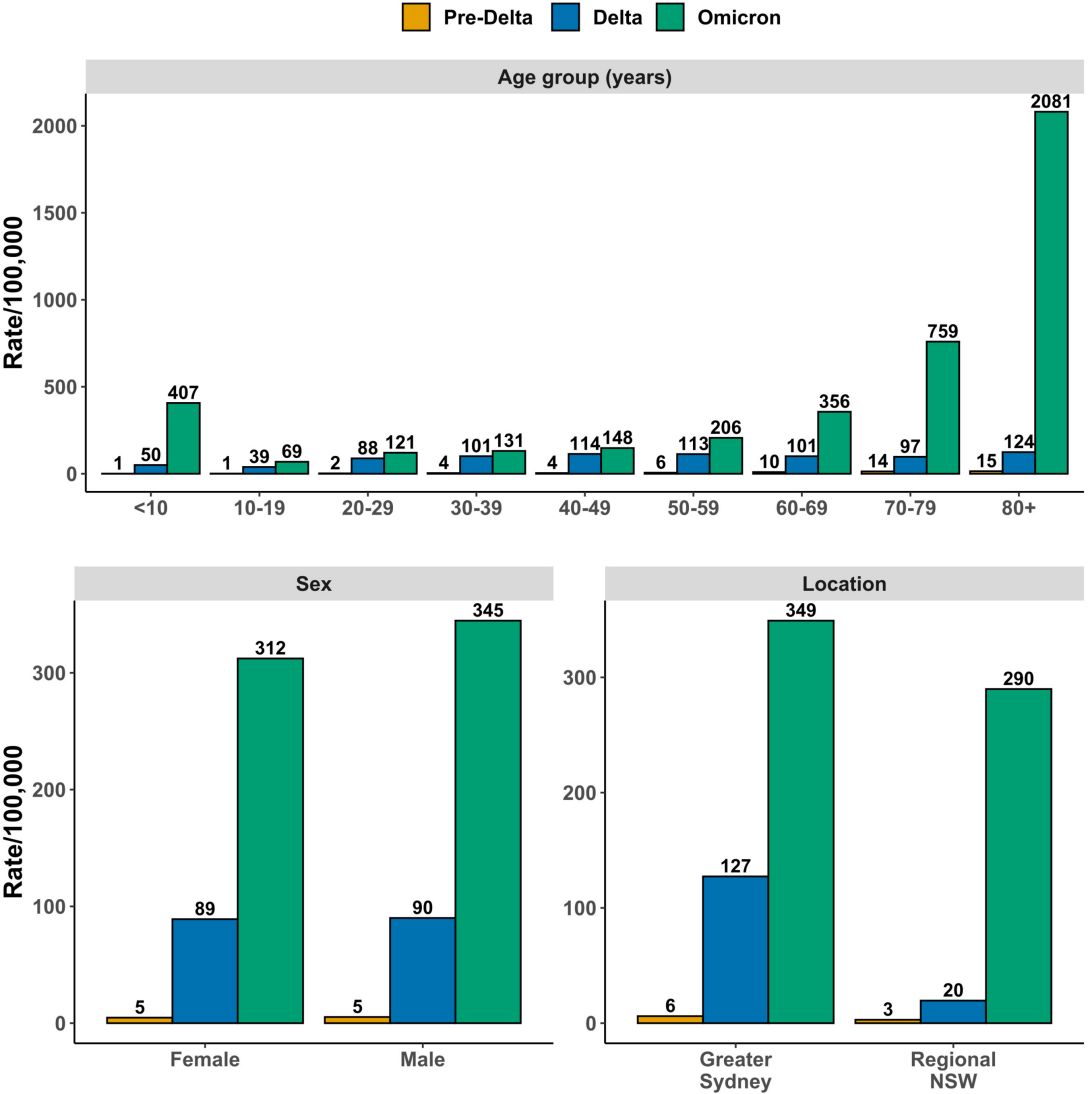
Incidence per 100,000 population of inpatient admissions within 28 days of index ED arrival with a linked SARS‐CoV‐2 infection, by age group, sex, and location of residence.

The incidences of inpatient admissions following index ED presentations were 5, 90, and 328 per 100,000 population during the pre‐Delta, Delta, and Omicron phases, respectively. During the pre‐Delta phase, < 10‐year‐olds and 10–19‐year‐olds had the lowest incidence of inpatient admission at 1 per 100,000, but incidence gradually rose with age to 15 per 100,000 in ≥ 80‐year‐olds. During the Delta phase, < 10‐year‐olds had the second lowest incidence at 50 per 100,000, with the lowest being in 10–19‐year‐olds at 39 per 100,000. In older age groups during the Delta phase, incidence of inpatient admission was similar, ranging from 88 to 124 per 100,000. During the Omicron phase, the highest incidence was among ≥ 80‐year‐olds (2081 per 100,000), followed by 70–79‐year‐olds (759 per 100,000), and < 10‐year‐olds (407 per 100,000) (Figure 3).

During the Omicron phase, the incidence of inpatient admissions among females was slightly lower than that of males (312 vs. 345 per 100,000 population), despite being comparable during the pre‐Delta and Delta phases. Throughout all three epidemic phases, the incidence of inpatient admissions was consistently higher among Sydney residents compared to those in regional NSW. The most substantial difference in inpatient admission incidence was observed during the Delta phase with 127 per 100,000 among Greater Sydney residents compared to 20 per 100,000 among regional NSW residents (Figure 3).

## 4 Discussion

In this record linkage study, we found that the pre‐Delta phase in NSW was characterized by a very low relative incidence of COVID‐19 associated ED presentations (13 index presentations per 100,000) and inpatient admissions (5 per 100,000) compared with later phases, with only a slight increase with increasing age. The proportion of children aged under 10 years among ED presentations and inpatient admissions increased with each phase, reaching 23% and 15%, respectively, in the Omicron phase.

The briefer Delta phase had a higher incidence of ED presentations (117 index presentations per 100,000) and inpatient admissions (90 per 100,000) compared to pre‐Delta, and was characterized by the highest proportion of ambulance transports (74% versus ≤ 30%), inpatient admissions (76% versus ≤ 39%), and ICU admissions (12% versus ≤ 9%) of any phase.

The Omicron phase to the end of 2022 was characterized by much higher incidence of ED presentations, with one index ED presentation for every 100 persons of any age presenting with COVID‐19. This period was also characterized by relatively increasing prominence of < 10 and ≥ 80‐year‐olds presenting to ED, with a 28‐fold and 20‐fold increase in incidence of index ED presentation, respectively, relative to the Delta phase. This compared with a 9‐fold increase in all ages combined. For inpatient admission, the respective increases in < 10‐year‐olds, ≥ 80‐year‐olds and all ages were 8‐fold, 17‐fold and 4‐fold. During the Omicron phase, nearly 3 in 100 persons aged ≥ 80 years had at least one index ED presentation and 2 in 100 in the same age group had an inpatient admission. Regional NSW residents were also more prominent during the Omicron phase with them representing 36% of presentations compared with ≤ 18% in earlier phases, indicating more virus transmission in the Sydney metropolitan region during earlier phases. The proportion of index ED presentations with an inpatient (33%) or ICU (2%) admission was lowest during the Omicron phase.

The substantial decline in median age of ED presentation and inpatient admission from the pre‐Delta to Delta phase may be linked to a combination of interventions including the prioritization of vaccination for older people and restrictions imposed on visiting aged care facilities [22]. As the COVID‐19 epidemic in NSW progressed, there was a disproportionate increase in the incidence of children < 10 years and older adults aged ≥ 80 years presenting to the EDs and being admitted to hospital for inpatient care.

The higher incidence of ED presentation and inpatient admission in < 10‐year‐olds during the Omicron phase was likely due to the variant's high transmissibility among children, relatively low coverage of vaccination in this age group, or coincidental infection in children admitted for other reasons [23, 24, 25], although we only included patients with acute respiratory infection‐like ED diagnoses. COVID‐19 vaccination of children aged 5–11 years commenced in early January 2022, as Omicron was starting, and by the end of that year, 39.1% of that age group had received two doses [26]. COVID‐19 vaccination for children below 5 years of age and healthy children between 5 and 17 years of age is not recommended in Australia. Severe outcomes after inpatient admission, such as ICU admission, ventilation, or death, were rare among this age group [13]. The only comparable Australian study reported that less than 1% of children (< 16 year) with COVID‐19 infection required inpatient admission [7], but that data only included the Delta phase, June 1–October 31, 2021, and only included children living in areas served by three of six metropolitan Sydney local health districts.

Low incidence of ED presentation with COVID‐19 in NSW during the pre‐Delta phase is consistent with the findings from a seroprevalence study in Sydney conducted after the first epidemic wave of 2020, which documented that < 1% of the population had antibodies against SARS‐CoV‐2 [27]. Initially the epidemic was contained by public health measures including international and interstate travel restrictions, hotel quarantine, movement restrictions, density restrictions, and mandatory mask use [22, 28]. This became more difficult in subsequent phases due to the emergence of more infectious variants of the SARS‐CoV‐2 virus and increased community transmission following the gradual easing of nonpharmaceutical interventions from late 2021, which coincided with the emergence of the Omicron variant [17].

The finding of a high proportion of ED presentations with ambulance transport or inpatient admission during the Delta phase aligns with previously reported increased severity of Delta over the pre‐Delta and Omicron variants of the SARS‐CoV‐2 virus [29, 30, 31]. Stay‐at‐home orders during the Delta phase may have contributed to increased ambulance use if patients were concerned about leaving their home to attend hospital.

Among those admitted for inpatient care, ICU admission and ventilation support decreased by 75% and 83%, respectively, from the pre‐Delta to the Omicron phase. This would be linked to increasing vaccine coverage in the state, reduced severity of the Omicron relative to the Delta variant, and the vaccine's efficacy in preventing severe outcomes following infection with the virus [32, 33]. By the end of the pre‐Delta phase (May 2021), about 15% of the eligible population at the time, who were persons aged at least 50 years, had received a first dose of the COVID‐19 vaccine. By late November 2021 (end of Delta phase), > 91% in Greater Sydney, and > 94% in regional NSW, of ≥ 16‐year‐olds had received two doses. By the end of the study period (December 2022), 2‐dose vaccine coverage in the same age group reached 96%, and was 67% for the third dose [34]. The effectiveness of the vaccine in preventing severe outcomes in NSW during the Delta phase is shown by the low proportion, relative to the population vaccine coverage levels, of fully vaccinated individuals among patients with inpatient or ICU admission, or death, observed, in active surveillance reports [18]. The vaccine continued to be effective during the Omicron phase [35].

The proportion of inpatients that died was at most 7% in all three phases and did not vary markedly over the study period in NSW. In contrast, other regions reported higher mortality rates among hospitalized patients, with England having mortality as high as 40.3% in March 2020 to below 10% in March 2021 and a meta‐analysis reporting a rate of 16% [36, 37]. Older patients are at greater risk of a severe outcome, and the median age of inpatients rose from 45 to 68 years from the Delta to the Omicron phase [38, 39]. Thus, all other things being equal, deaths would have been more frequent as a result of the age increase, but this did not eventuate.

A limitation of the study is that the reporting of COVID‐19‐related ED presentations and hospitalizations was influenced by testing behavior, which was less comprehensive during the Omicron phase compared to the pre‐Delta and Delta phases [40]. However, our analysis spans a longer period than previously published data, encompassing multiple COVID‐19 waves and enabling a comprehensive characterization of the evolving epidemic in NSW. Additionally, incidence rates may be slightly underestimated because we did not have access to data for patients receiving care from health services in adjacent states or territories. We also lacked data from one public and the few private hospitals in the state.

In conclusion, the study highlights the epidemiological shifts occurring with each of the three phases of the COVID‐19 pandemic in NSW, from its onset in 2020 to the end of 2022. These changes include a dramatic rise in ED presentations when the Omicron variant arrived, one presentation per 100 persons, with higher rates in persons aged ≥ 70 and < 10 years. With Omicron, there was an increased spread of the virus to regional areas. We provide further evidence that the Omicron variant presents an increased risk of inpatient admission in children compared with earlier variants. While < 5 deaths occurred in < 10‐year olds, the increased risk of inpatient admission due to infection with the Omicron variant justifies continued monitoring of the safety and effectiveness of the COVID‐19 vaccine in that age group and should be considered in the development of COVID‐19 vaccination recommendations. The low proportion of deaths occurring among inpatients compared with other countries during all three variant phases is consistent with high‐quality healthcare and effectiveness of both nonpharmaceutical and vaccination measures implemented in the state in limiting virus transmission to vulnerable age groups, especially older persons.

## Author Contributions

K.K.P. and D.J.M. conceived and designed the study; K.K.P., N.R., and D.J.M. cleaned, processed, and performed the analysis, K.K.P. drafted the manuscript, N.R., S.W., M.M.D., and D.J.M. aided in interpreting the results and provided feedback on the manuscript. D.J.M. supervised the study. All authors approved the results and agreed to publish the manuscript.

## Funding

This research work was supported by an Australian National Health and Medical Research Council (NHMRC) Investigator Grant (APP1194109) to DJM. The contents of the published material are solely the responsibility of the Administering Institution, a Participating Institution or individual authors and do not reflect the views of the NHMRC.

## Ethics Statement

The study was approved by the NSW Population and Health Services Research Ethics Committee (2021_ETH00070).

## Conflicts of Interest

The authors declare no conflicts of interest.

## Data Availability

The data that support the findings of this study are available from the NSW Centre for Health Record Linkage. Restrictions apply to the availability of these data, which were used under license for this study. Data are available from the authors with the permission of the Centre for Health Record Linkage.
